# Mechanisms Underlying the Interaction Between Chronic Neurological Disorders and Microbial Metabolites *via* Tea Polyphenols Therapeutics

**DOI:** 10.3389/fmicb.2022.823902

**Published:** 2022-03-25

**Authors:** Mengyu Hong, Lu Cheng, Yanan Liu, Zufang Wu, Peng Zhang, Xin Zhang

**Affiliations:** ^1^Department of Food Science and Engineering, Ningbo University, Ningbo, China; ^2^Department of Food Science, Rutgers, The State University of New Jersey, New Brunswick, NJ, United States; ^3^Department of Student Affairs, Xinyang Normal University, Xinyang, China

**Keywords:** tea polyphenols, intestinal metabolites, host health, chronic brain diseases, interaction

## Abstract

The number of hydroxyl groups and existence of characteristic structural groups in tea polyphenols (TP) make them have antioxidant activity, which gives TP anti-inflammatory effects, toward protecting the intestinal flora and brain neurons. Host-associated microbial metabolites are emerging as dominant modifiers of the central nervous system. As yet, the investigations on host-microbiota crosstalking remain challenging, studies focusing on metabolites such as serotonin, short-chain fatty acids, and others have pinpointed multiple actionable signaling pathways relevant to host health. However, there are still complexities and apparent limitations inherent in transforming complex human diseases to corresponding animal models. Here, we choose to discuss several intestinal metabolites with research value, as crucial areas for assessing TP-mediated chronic brain diseases interactions with microbial.

## Introduction

Tea, coffee, and cocoa are collectively known as the three major beverages in the world. According to the different fermentation levels of contemporary tea, people divide tea into six categories, namely green tea, white tea, yellow tea, oolong tea, black tea, and dark tea. Green tea and white tea belong to unfermented tea. Green tea contains a variety of substances, such as tea polyphenols (TP), proteins, amino acids, vitamins and others (Han et al., 2016). Yellow tea is lightly fermented, oolong tea is semi-fermented, black tea is fermented and dark tea is post-fermented (Zhu et al., 2020).

Tea contains a wide range of compounds, but the substances with antioxidant and beneficial effects are mainly polyphenols (PPs). PPs are a large group of phytochemicals, containing one or more hydroxyl aromatic rings. They exist as secondary plant metabolites in most fruits and vegetables, as well as herbs and spices (Saric et al., 2016). PPs have attracted much attention for their anti-cancer, antioxidant, antibacterial, anti-inflammatory, and prevention of chronic diseases such as diabetes, obesity, neurodegenerative diseases and cardiovascular diseases (Ohishi et al., 2021). PPs with higher molecular weight, due to their chemical complexity, will not be absorbed in the small intestine. It has been proven that only 5–10% of the total intake of PPs can be absorbed by the small intestine, which depends largely on their structure and combination with sugar moieties (Zhou et al., 2020).

Tea catechins account for about 70–80% of the total PPs. Catechins belong to flavanols and are a derivative of 2-phenylbenzopyran. Catechins mainly include (−)-epigallocatechin-3-gallate (EGCG), (−)-epicatechin (EC), (−)-epigallocatechin (EGC) and (−)-epicatechin-3-gallate (ECG). Among them, the content of EGCG is the most abundant, accounting for about 50 ∼ 80% of the total catechins. Growing evidence suggest that EGCG reshapes gut microbiota architecture. Remely et al. (2017) stated that EGCG interventions reduced *Clostridium* spp., increased *Bacteroides*, and altered propensity of *Bifidobacterium* and *Prevotella* in the murine gut. Research is currently underway to reveal that the microbiota can impact the brain through the microbiota-gut-brain (MGB) axis (Sampson et al., 2016). Understanding how this MGB axis communication may lead to disease and homeostasis in the body is the key to human health and well-being. Consistent with observations those neurological diseases patients are conventionally accompanied by intestinal symptoms, their microbial compositions are distinct from healthy participants, since microbial transplantation alters the disease pathophysiology (Jameson et al., 2020). Intestinal flora has some relations with neurological diseases, wherein gut dysbiosis reduce the rescue effect of EGCG. Therefore, the intestinal microbial structure is not only related to the progression of a series of body diseases, but also mediates the nutritional intervention of active compounds *in vivo*.

5-hydroxytryptamine (5-HT) is a brain-gut peptide and neurotransmitter widely distributed in the central nervous system (CNS) and gastrointestinal (GI) tract. Its anabolic and physiological functions are regulated by the intestinal flora. Intestinal flora can regulate the strength of intestinal motility by interfering with the balance of 5-HT in the intestine (Reynaud et al., 2016). Both of them play an important role in the pathogenesis of intestinal diseases and CNS diseases such as inflammatory bowel syndrome (IBS), Alzheimer’s disease (AD) and depression (Fung et al., 2019).

Henceforth, in this contribution, we discuss the overview of TP-mediate for the effective treatment of CNS disorders and intestine-related diseases. As an essential neurotransmitter, 5-HT plays a significant role in the signal pathway of the MGB axis. It is important to note, this complex interaction between TP, intestinal flora, 5-HT, and brain seem to underlie the development of intestinal inflammation, psychiatric and neurodevelopmental disorders, and may inform the safe use of TP therapy.

## Tea Polyphenols and the Biochemical Properties

**FIGURE 1:**
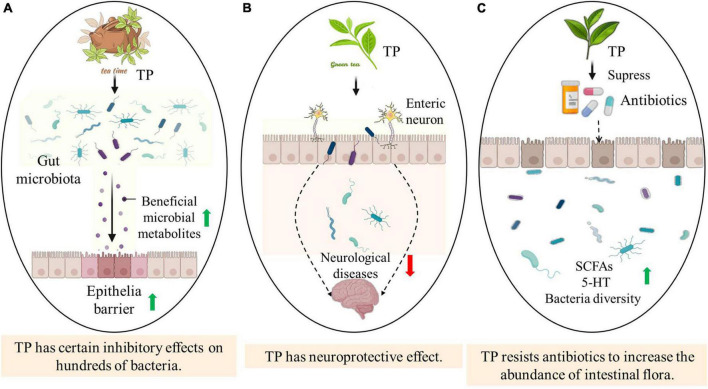
The impact of the multiple biodiversity of tea polyphenols on the host. Panel **(A)** describes that when tea polyphenols is ingested by the host, tea polyphenols interacts with intestinal flora, thereby increasing some beneficial metabolites that enhance the epithelial barrier and thus resist pathogenic bacteria. Panel **(B)** shows that TP has a certain regulatory effect on the enteric nervous system, so as to prevent, cure or at least alleviate neurological diseases. As shown by Panel **(C)**, TP significantly increases the richness and diversity of intestinal flora after antibiotic treatment, improves the imbalance of intestinal flora, and increases the level of SCFAs and 5-HT.

Tea polyphenols is a general term for a class of phenolic compounds contained in tea. A large number of experimental studies *in vitro* have shown that TP has strong antioxidant activity (Musial et al., 2020). The antioxidant function of individual TP depends on its molecular structure, the position of the hydroxyl group and other substituents (Yan et al., 2020). TP can provide active hydrogen through hydroxyl to generate more stable phenolic free radicals to scavenge active oxygen and free radicals. TP displays the ability of broad-spectrum and strong antibacterial properties, and certain inhibitory effects on hundreds of bacteria in nature (Wang et al., 2020). There are many metal ions in the bacteria, some of which are coenzymes and others are essential elements. TP with polyhydroxyl structure can react with iron, calcium and other metal ions to form cyclic chelates, resulting in the formation of precipitation, thus affecting bacterial enzymes, activity and the growth and reproduction of sensitive microorganisms (Chen et al., 2016). The phenolic hydroxyl group and benzene ring in the structure of TP can also form hydrogen bonds or hydrophobic structures with proteins, which affect the normal expression of proteins, thereby inhibiting the activity of bacteria. Studies have also shown that the antibacterial effect of TP is related to its degree of polymerization. Compared with the monomers of TP, its oligomers show stronger antibacterial properties (Sasaki et al., 2004) (Figure 1A).

In addition to anti-oxidative, scavenging free radicals, chelating metal ions, anti-cancer, anti-inflammatory and anti-apoptotic properties, TP also has potential therapeutic potential in preventing neurodegenerative diseases (Yan et al., 2021). A large number of studies have reported that TP possesses a protective effect against brain damage in a variety of animal models of Parkinson’s disease (PD) (Liu et al., 2013). TP extract and monomer EGCG can reduce striatal dopamine consumption and substantia nigra dopaminergic neuron apoptosis. Abundant animal models indicate that TP such as EGCG and EC can pass through the blood–brain barrier. After the rats ingested EGCG (500 mg⋅kg^–1^), the concentration of EGCG in the brain reached 0.5 nmol⋅g^–1^ and localized in the brain tissue (Sun et al., 2021). These findings indicate that TP have potentially bioactive substances with neuroprotective and neuromodulating effects (Figure 1B).

The antioxidant effect of TP may protect the dopamine system against free radicals, anionic superoxide and intracellular hydroxydopamine-induced neuronal apoptosis (Khan and Mukhtar, 2018). In the CNS, TP can inhibit lipid oxidation and the accumulation of divalent iron complexes, which may be the main mechanism of its neuroprotective effect. The inhibitory effect of TP on lipid peroxidation includes the scavenging of inorganic free radicals. First, TP participates in the initial reaction, effectively scavenging reactive oxygen species such as oxygen ions, and preventing the initiation of lipid peroxidation; secondly, it reacts with lipid free radicals of lipid peroxidation, resulting in chain breakage. The combination of these two effects made TP show significant anti-lipid oxidation function (Tian et al., 2021). In mitochondria, TP may stimulate cellular energy expenditure, thereby reducing weight gain. At the same time, in the nucleus, TP may inhibit the expression of fatty acid synthase gene by down-regulating EGF-receptor/PI3K/Akt/AP-1 signal transduction pathways, thus inhibiting blood lipids and cell growth (Lin and Lin-Shiau, 2006). EGCG is an important antioxidant and iron chelating substance (Zhang et al., 2021). The 3′, 4′-dihydroxy group and gallate group in the B-ring may reduce the divalent iron ions to inactive iron atoms and thus protect cells from oxidative stress damage. EGCG inhibits more than 90% of DNA damage mediated by chelating metal ions (Perron et al., 2008, 2010). Therefore, the protective effect of TP is partly due to free radical scavenging or metal chelation.

## The Essential Roles of Intestinal Microbiota on the Body

Humans can be divided into three different “enterotypes” based on the type and number of bacteria in the intestine. Researchers named these three enterotypes as *Bacteroides*, *Prevotella*, and *Ruminoccus* type to reflect the dominant bacteria in each ecosystem (Romo-Vaquero et al., 2019). The bacteria in the *Bacteroides* type system mainly obtain energy from carbohydrates and proteins; the *Prevotella* system is good at digesting glycoproteins in the intestines, and this tendency is the same as the rumen cocci system (Costea et al., 2018). Although the knowledge of enterotypes is far less than the understanding of blood type, scientists believe that the information of enterotypes can also provide a reference for the diagnosis and treatment of diseases. The type and number of flora in the intestine reflect the digestive ability, immune ability and response of different people to drugs.

Each species or strain of gut mutualistic bacteria performs unique biological functions, and therefore, their balance is essential for maintaining GI homeostasis. Importantly, through cooperation and competition, the colonization of each type of bacteria restricts and influences each other (Fassarella et al., 2021). Different types or strains of symbiotic bacteria in the intestine harbor different nutritional preferences and are used to adapt to the intestinal environment.

Furthermore, another species of *Lactobacillus* named as *Lactobacillus plantarum* can be used as probiotics, which contributes to the host health. For example, *L. plantarum* has beneficial effect against metabolic syndromes, diabetes, and brain diseases (Liu Y. W. et al., 2018). Wang et al. (2013) found that *L. plantarum* strain ZLP001 could fortify the intestinal barrier function by reinforcing the intestinal epithelium and modulating gut microbiota composition. In addition, the probiotics contained in healthy microbiota can elicit host protective immunity or anti-inflammatory immunity, thereby helping to combat pathogens and promote the recovery of inflammatory damage (Guo et al., 2020). Rats prone to diabetes were fed with *Lactobacillus johnsonii* N6.2 and/or rosmarinic acid. After a period of time, it was found that the early gastrointestinal inflammation in rats was effectively alleviated. As a potential probiotic, *L. johnsonii* N6.2 can reduce the level of pro-inflammatory cytokines, such as interleukin (IL)-1β and lower levels of interferon (IFN)-γ transcription (Teixeira et al., 2018).

Studies have observed that alterations in intestinal flora are capable of promoting the occurrence and progression of chronic diseases, and the progress of chronic diseases can also aggravate the disorder of intestinal flora (Liu et al., 2010). The bacteria in the intestine are mainly composed of *Firmicutes* (F) and *Bacteroides* (B). The ratio of F/B in the intestine not only affects carbohydrate metabolism, but also alters the production of short-chain fatty acids (SCFAs). There are strong tight junction proteins between the intestinal epithelium of normal people, which connect the intestinal epithelial cells together. However, high blood sugar in diabetic patients can elicit intestinal barrier damage, leading to microbial metabolites entering the body, promoting the proliferation, transferring of pathogenic bacteria, and worsening intestinal infection status (Gong et al., 2017).

Intestinal flora dysregulation is not narrowly defined as a disorder of bacteria in the intestinal tract, and also includes conversions of fungi and viruses in the bowel (Elson and Cong, 2012). Li et al. (2018) demonstrated that intestinal fungal disorders caused by anti-fungal treatment or inoculation of some typical rare fungi would cause excessive immune response, which illustrated the significant role of intestinal fungi in immune homeostasis. Research by Sartor and Wu (2017) indicated specific changes in the intestinal fungi of Crohn’s disease, which implicated that the unique intestinal environment of Crohn’s disease might be instrumental in fungi, whereas impaired the growth and reproduction of bacteria and promoted the perturbation of intestinal flora. Accordingly, the disorder of the intestinal flora is a complex, including different degrees and types of dysfunction forms. When studying chronic diseases and intestinal flora disorders, it should not be simply considered as alterations in the structure and richness of bacteria, which also includes flora metabolism, fungi, viruses, and others.

## Gut Microbiota-Derived Metabolites and Their Effects on Host Health

In the past 15 years, scientists have proved that gut microbes are involved in nutrient absorption, substance metabolism, immune defense and other important physiological processes, associated with a variety of diseases, such as diabetes, heart disease, allergy and depression (Qi et al., 2021). Metabolism is the common basis of all cellular processes involved in the host, host microbiota, and invading pathogens. One of the main ways for intestinal flora to interact with host is through metabolites, which are intermediate or final products of microbial metabolism (Troha and Ayres, 2020). These metabolites are derived from dietary digestion, modification of host molecules, such as bile acids, or directly from bacteria. These metabolic signals affect immune maturation, immune homeostasis, host energy metabolism and mucosal integrity. One of the reasons why these tiny organisms have such a big impact is that they secrete metabolites into the blood circulation (Oliphant and Allen-Vercoe, 2019). The existence of MGB axis makes the intestinal tract and brain of the host communicate closely. MGB axis functional changes are involved in the occurrence of a variety of gastrointestinal diseases, such as IBS and related functional gastrointestinal diseases. Studies have also found that MGB axis disorders are also involved in many brain diseases, including autism, PD, depression and chronic pain. Intestinal microorganisms and their metabolites can regulate gastrointestinal function by affecting intestinal permeability, mucosal immune function, gastrointestinal motility and enteric nervous system (ENS) activity; intestinal microorganisms and their metabolites are also involved in regulating behavior and brain processes, including stress response, emotional behavior, pain regulation, feeding behavior and brain biochemistry (Raskov et al., 2016; Liang et al., 2018).

Host can directly detect the metabolites of microorganisms and coordinate the process and physiology of the host. The intestine of a mammal consists of a single epithelial layer that physically isolates the microbiota from the rest of the body and prevents excess solutes, microorganisms and lumen antigens from entering the body (Laukoetter et al., 2008). Intestinal epithelium directly senses microbial metabolites and induces the host’s response, further promotes the integrity of the barrier. For example, in order to maintain a homeostatic relationship with the intestinal microbiota, the barrier integrity of the intestinal epithelial layer must be maintained (McCarville et al., 2020). Intestinal barrier includes physical barrier, chemical barrier, microbial barrier and immune barrier. The epithelial cell layer and the internal and external mucin layer constitute the physical barrier, while the mucus, digestive juice produced by the epithelial cells and the bacteriostatic substances secreted by normal bacteria are chemical barriers, and the mucosal flora and intestinal microflora form a multi-level intestinal microbial barrier. Intestinal associated lymphocyte tissue and diffuse immune cells form immune barrier (Ghosh et al., 2020). If the intestinal barrier is broken, lipopolysaccharide (LPS) is transported through the blood to LPS-binding proteins or lipoproteins, and CD14 as a cofactor interacts with surface receptors on immune cells, such as toll-like receptor 4, to initiate inflammatory response, which in turn activates NF-κB pathway, resulting in increased transcription of pro-inflammatory cytokines such as tumor necrosis factor (TNF)-α, IL-1β and IL-6 (Lu et al., 2008).

**FIGURE 2:**
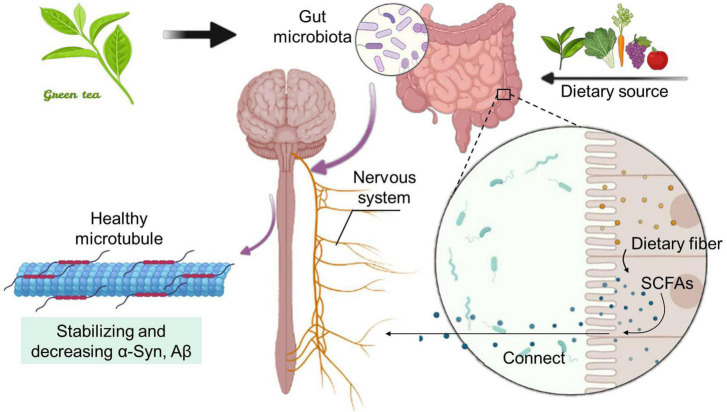
Dietary fiber-produced SCFAs and tea polyphenols-mediated intestinal flora work together to establish a connection with the host nervous system. The intake of TP and dietary fiber can increase the amount of SCFAs, a metabolite in the intestinal tract. The pathway between the intestinal tract and the brain makes SCFAs as a transmission medium, which can effectively improve neurological diseases.

The SCFAs are organic fatty acids with carbon chain length less than 6, mainly including acetic acid, propionic acid, butyric acid and valeric acid, and is one of the metabolites with the highest microbial content in the intestinal lumen (Zhao et al., 2016). Studies have shown that the use of probiotics *Lactobacillus johnsonii* N6.2 and phenols may be an effective way to improve metabolic syndrome related diseases caused by high-fat diet, help to maintain the healthy and steady state of the intestinal tract, and thus promote the metabolic production of SCFAs (Teixeira et al., 2021). SCFAs can enhance the health of the host by reducing inflammation, improving autoimmune diseases and allergies, maintaining the intestinal barrier and mediating the colonization resistance of intestinal pathogens to enhance the health of the host and regulate the functions of multiple systems, such as the intestines, nerves, endocrine and blood systems (Overby and Ferguson, 2021). A large amount of evidence shows that SCFAs plays a vital role in maintaining intestinal health, preventing and improving a variety of non-communicable diseases, including cancer, and are one of the most important intermediates between disease, nutrition and intestinal flora (Van Treuren and Dodd, 2020). As an essential medium, it directly or indirectly plays an important physiological role in multiple organs and tissues of the body (Figure 2). More and more studies have shown that the SCFAs produced by dietary fiber under the action of microflora can act not only on the intestinal tract, but also on the distal part of the brain. As one of the receptors of SCFAs, Gpr41 is widely expressed in the peripheral nervous system in addition to ENS, such as sympathetic ganglia, vagus nerve, dorsal root and trigeminal ganglia. One of the effects of SCFAs on the brain is that during intravenous administration, a small amount of acetic acid is absorbed across the blood-brain barrier and activates hypothalamic neurons, resulting in a sense of satiety (Koh et al., 2016; Makki et al., 2018).

**FIGURE 3:**
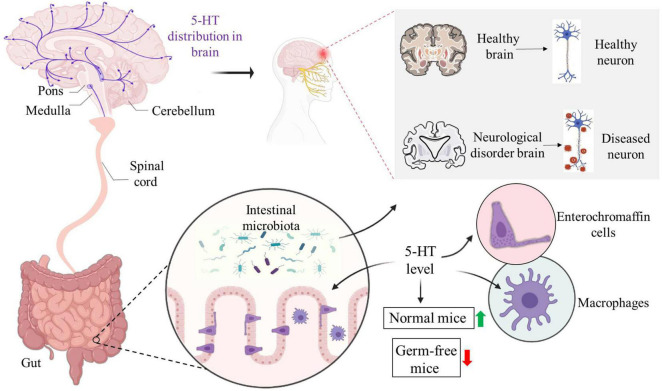
Distributions of 5-HT in the brain and intestine affect the brain nerves. The distribution of 5-HT in the brain affects the state of the brain, which is different between health and neurological diseases. Enterochromaffin cells and macrophages can promote the secretion of 5-HT in the intestine. Compared with normal flora colonized mice, the level of 5-HT in germ-free mice was lower. The level of 5-HT may also regulate the intestinal microecological environment, which can affect the function of the central nervous system and is associated with neurological diseases.

Tryptophan is an important amino acid, which affects the metabolism of the host through metabolites produced by the three main fermentation pathways of the intestinal flora and GI cells (Keszthelyi et al., 2009). Tryptophan can be broken down by the intestinal flora into indole and its derivatives called aryl hydrocarbon receptor ligand; it is metabolized by the kynurenine pathway in immune and epithelial cells, and its activity is regulated by the gut flora; it also produces 5-HT by tryptophan hydroxylase 1 in enterochromaffin cell (Gheorghe et al., 2019). Since animal cells cannot produce tryptophan, humans rely on exogenous, mainly dietary intake, including bananas, milk, peanuts and others. The tryptophan metabolism pathway has been identified in the intestinal flora of some people, such as *Clostridium sporogenes*, which can achieve decarboxylation and lead to the production of the neurotransmitter tryptamine. Tryptamine (a tryptophan metabolite produced by *C. sporogenes* and *Ruminococcus gnavus*) is a β-arylamine neurotransmitter that is involved in intestinal health (Jenkins et al., 2016). In the intestine, tryptamine induces the release of the neurotransmitter 5-HT through enterochromaffin cells (ECs) located on the mucosal surface, and 5-HT stimulates GI motility by means of neurons in the enteric nervous system (Okaty et al., 2019). 5-HT is an inhibitory neurotransmitter with high content in the cerebral cortex and nerve synapses (Figure 3). It can enhance memory and protect neurons from “excitatory neurotoxin” damage and is generally considered to be a contributor to happiness.

## Influences of Intestinal Bacteria Disorder on the Body

The perturbation of this balance “so-called gut dysbiosis” triggers or exacerbates various GI diseases, such as inflammatory bowel diseases (IBD). There is also ample evidence that dysbiosis can increase intestinal inflammation. For example, a decrease in strict anaerobes and an increase in facultative anaerobes lead to increased intestinal inflammation (David et al., 2014). In addition, decreased numbers of SCFA-producing microorganisms, such as *Clostridium* spp. has been observed in IBD patients (Nagao-Kitamoto and Kamada, 2017). Microbial-derived SCFAs, especially butyrate, promote the production of regulatory T cells and intestinal mucus to block inflammatory signal transduction pathways and strengthen the epithelial barrier function (Thibault et al., 2010). Another feature of the IBD microbiome is the reduction of tryptophan metabolism. The tryptophan metabolite indole acrylic acid produced by several species of *Peptostreptococcus* spp. promotes mucosal barrier function and reduces inflammation (Nikolaus et al., 2017). Intestinal inflammation caused by pathogens significantly changes the intestinal microenvironment, which in turn affects the adaptability and stability of GI bacteria, thus forming a structure of resident microbial communities.

Factors involved in the development of an unhealthy state of the gut microbiome include drastic changes in dietary patterns, microbial infections and frequent use of medications, especially antibiotics (Matsuoka and Kanai, 2015). External disturbances can cause the stable microbial ecosystem to be pushed into an unstable state, after which it may return to its original state. Nevertheless, they may also lead to another healthier and more stable state. A stable microbial community can resist the invasion and expansion of non-native bacteria and pathogens, resulting in the phenomenon known as colonization resistance (Ding and Schloss, 2014). The interactions between the host and the gut microbes need to actively maintain a dynamic equilibrium in order to achieve a healthy and stable state.

## Tea Polyphenols and the Beneficial Effects on Intestinal Microbiota

The regulation of TP on the composition and function of the intestinal flora was generally dose-dependent, and different doses of TP could dramatically change the relative abundance of *Lactobacillus* after antibiotic action. Low-dose TP (daily dose of 90 mg/kg) apparently antagonized the decrease in the abundance of *Lactobacillus* caused by antibiotics, even significantly higher than the normal group (only received distilled water) (Li et al., 2021). However, the influence of TP on the intestinal microflora depended on the microbial strains, TP structure and testing dose. High doses of TP (daily dose of 360 mg/kg) might adversely affect probiotics, leading to inactivation of *Lactobacillus* (Li et al., 2021). Because excessive intake of TP may cause damage to the liver of animals, produce certain toxic side effects, and the probiotics will also be affected (Patel et al., 2013). Therefore, in order to avoid the possible negative effects of high-dose TP on the intestinal flora and maintain the balance of the intestinal flora, the intake of TP at an appropriate dose probably plays an effective role.

Antibiotics have been used to treat or alleviate certain diseases, nevertheless, antibiotics tremendously alter the composition and metabolic activity of the intestinal microflora (Kim et al., 2017). TP significantly increases the richness and diversity of the gut microflora after antibiotics treatment, improves the imbalance of the intestinal microflora, increases the level of SCFAs, and reduces the risk of cancer, obesity, diabetes and other diseases caused by antibiotics (Pérez-Burillo et al., 2021). The modulation of intestinal microflora by TP may be one of the mechanisms of its anti-tumor, anti-obesity, immune regulation and other biological activities. Accordingly, TP has a potential to be a functional food additive to reduce the negative effects of antibiotics (Gowd et al., 2019) (Figure 1C).

Several types of studies have shown that TP can regulate the intestinal microbial community by exerting a probiotic-like effect or antibacterial activity on intestinal pathogenic bacteria (Zhang et al., 2020). TP has been considered as a natural source of antibacterial agents, which can inhibit the growth of some pathogenic bacteria such as *Escherichia coli* (*E. coli*), *Streptococcus, Bacteroides*, and *Parasutterella* (Liu Z. et al., 2018). TP can inhibit the production of pro-inflammatory factors and reduce the incidence of colorectal cancer by decreasing the synthesis of bacterial lipopolysaccharide and the abundance of functional pathways related to cancer (Huang et al., 2021). By increasing the abundance of *Akkermansia* and butyric acid production, the intestinal flora composition and metabolism of EGCG-mediated colitis mice were significantly changed, and the symptoms of colitis were alleviated. After oral administration of EGCG, the levels of proinflammatory cytokines IL-6, IL-1β, and TNF-α in colon decreased significantly, the level of antioxidation increased, and eight kinds of probiotic genera such as *Bifidobacterium* and *Faecalibaculum* were enriched (Wu et al., 2021). Various researches also reported about the bioavailability of PPs and its impact on the host, which depended on their biotransformation into specific compounds by the action of gut microbiota (Cardona et al., 2013; Duda-Chodak et al., 2015; Roopchand et al., 2015). In the rat intestinal microflora, bacteria such as *Enterobacter aerogenes* and *Raoultella planticola* can decompose the chemicals in tea and then be absorbed by the body. And then enrich *Faecalibacterium*, *Bifidobacterium*, *Lactococcus*, *Coprococcus*, and other bacteria in the intestinal tract (Gan et al., 2018).

Following the transformation, the TP biological activity can be enhanced. The human small intestine cannot fully absorb TP. Most of the TP is considered to be left in the intestine and converted into lactic acid type I metabolites (lactones, phenolic and aromatic acids, and simple phenols) and type II metabolites (glucuronate, sulfate, and oxymethyl derivatives). After which they are converted into intermediate metabolites propelled by colonic bacterial enzyme glycosylation, intestinal microbial dehydroxylation and demethylation (Zeng et al., 2019). There is further conversion into small molecular compounds, which enter the liver-intestine circulation or systemic circulation and exert various physiological functions, and finally metabolites are excreted from the body through urine or feces (Gowd et al., 2019).

## Chemical Signals Between the Intestine and the Brain

### Short-Chain Fatty Acids and Enteric Neurons

Intestinal microbes influence the brain and behavior through immune, neuronal and metabolic pathways. In particular, emerging evidence shows that certain members of the microbiota are able to synthesize and/or modulate many neurochemical substances whereby regulate neurotransmission, as well as many other metabolites that can directly or indirectly affect neuronal activity (Jameson et al., 2020). Neural pathways connect the intestines and the brain. Leading of these nerve pathways is the vagus nerve, which extends from the brainstem to the intestines and ENS (Strandwitz, 2018). The intestinal microbiota can help regulate the homeostasis and behavior of host through chemical communication with the nervous system (including direct and indirect signals) (Fassarella et al., 2021). As an example of direct signal transmission, SCFAs are lipids produced by intestinal microbes through fermentation of dietary fiber, emphasizing the close connection between the intestinal microbiota and nerve function (Morais et al., 2021) (Figure 2).

Although some animal experiments have emphasized microbial regulation of appetite is the basis for the difference in weight gain, we are very much in the dark how microbial regulation of SCFAs affects the host feeding behaviors. SCFA free fatty acid receptors 2 and 3 are expressed in the ENS, portal nerve and various sensory ganglia, prompting that activation of the nervous system plays a role in regulating these functions (Czajkowska and Szponar, 2018).

## Intestinal Microbiota Induce Neuronal 5-HT Production

5-HT, also known as serotonin, is approximately 95% derived from the gut and synthesized by the ECs and the myenteric plexus, while the remaining 5% is synthesized in central 5-HT neurons. Tryptophane in the intestine is catalyzed by tryptophane hydroxylase 1 in ECs to produce 5-hydroxytryptophan, which then tryptophan decarboxylase catalyzes the production of 5-HT (Rhoades et al., 2019).

In addition to being an important neurotransmitter, 5-HT is also a secretagogue, a crucial regulator for vast biofunctionals residing in the digestive tract, comprising the regulation seen in intestinal secretion and exercise. The majority of 5-HT are released from ECs, which not only regulate the diversified physiological functions in GI, but also have the function of modulating immunity and interacting intimately with mucosal immune cells (Li et al., 2016).

Immune cells are associated with various 5-HT receptors, including lymphocytes, monocytes, macrophages, T cells and B cells. 5-HT levels may be affected by gut microbes. In germ-free (GF) mice, the concentration of 5-HT was significantly reduced compared with the control group colonized by conventional flora. Spore-forming bacteria from healthy individuals promote the biosynthesis of 5-HT in colon ECs and then release it to the mucosa and lumen, thereby increasing the level of 5-HT (Meneses and Liy-Salmeron, 2012). One of the regulatory mechanisms of hypothalamic energy balance involves the 5-HT energy system. Its pharmacological stimulation can cause anorexia in humans and rodents, while inhibiting it increases food intake. Long-term consumption of high-fat foods will reduce postprandial insulin and 5-HT in the extracellular area of the hypothalamus, resulting in disorders of energy metabolism and increased inflammatory response. TP can improve high fat diet-induced hypothalamic inflammation, without affecting the 5-HT energy system (Okuda et al., 2014). 5-HT can change intestinal sensitivity and motility, and affect the pathophysiological process of IBS. Drugs acting on 5-HT receptors can relieve intestinal smooth muscle spasm, reduce visceral sensitivity, regulate intestinal motility, and improve abdominal pain and intestinal function in patients with IBS (Mikocka-Walus et al., 2020). Patients with IBS are usually accompanied by mental disorders, such as depression, anxiety and tension. People’s emotions are mainly controlled by the limbic system of the brain, which can regulate endocrine and autonomic nervous functions at the same time. Antidepressants act on the limbic system of the brain to improve mental state while relieving IBS symptoms (Macedo et al., 2017).

Furthermore, selected bacterial strains, such as *Bacteroides fragilis*, *Brucella* and altered *Schaedler* flora modify the levels of 5-HT in the colon and serum. *Corynebacterium*, *Streptococcus* and *E. coli* synthesize 5-HT through tryptophan *in vivo* (Nagao-Kitamoto and Kamada, 2017). Microbial mediation of alterations in 5-HT also has a regulatory effect on intestinal microenvironment in turn. Disturbance of the intestinal flora can cause imbalance of 5-HT levels, while the use of probiotics alleviates the symptoms of 5-HT dysfunction. Therefore, targeted bacteria can be used as a preferred method to regulate the bioavailability of peripheral 5-HT and treat disease symptoms (Jenkins et al., 2016) (Figure 3).

## Interactions Between the Microbiota and 5-HT are Neuroprotective

5-HT, as a major neurotransmitter, plays an important role in the MGB axis signaling pathway. The ENS is an irreplaceable and important hub in the physiological functions of the intestine, such as enterocinesia, secretion of digestive juice, intestinal blood flow, etc. (Furness, 2012). As we all know, the intestine contains trillions of bacteria, which modulate the host’s production of a variety of signal molecules including 5-HT, together with other hormones and neurotransmitters. 5-HT is widely present in mammalian tissues, with a high content in the synapses of the cerebral cortex, which can affect the human body’s mood, energy and memory. About 90% of 5-HT come from the intestinal tract. The activation of 5-HT_4_ receptor in the ENS is related to adult nerve formation and neuroprotection (Rebholz et al., 2018). 5-HT is a significant research substance, due in part to its diverse roles as a neurotransmitter in both the GI tract (that is, in processes such as peristalsis, secretion and absorption) and the CNS (that is, in regulation of pain modulation, sleep and mood) (Ochoa-Repáraz and Kasper, 2016).

5-HT is believed to play a non-negligible role in neurogenesis and improving the survival rate of nerve cells. De Vadder et al. (2018) confirmed that colonization of GF mice partially restored serum levels of 5-HT, likely by inducing *de novo* 5-HT synthesis by increasing the expression of the rate-limiting enzyme for 5-HT synthesis, *Tph1*, in the mucosa. Furthermore, depletion of the microbiota with antibiotics reduced circulating 5-HT levels.

Alzheimer’s disease, which represents the most common form of dementia in the elderly. It is clinically manifested as a large number of neuron reduction, brain structure atrophy, amyloid beta peptides (Aβ) deposition in the brain, senile plaque appears as well as neurofibrillary tangles (Włodarek, 2019). PD is a common neurodegenerative disorder along with midbrain substantia dopamine neuron necrosis in middle-aged and elderly people, huge reduced striatal dopamine, occurrence of α-Synuclein (α-Syn) aggregates in cells as well as formation of Lewy Body-predominant in neurons (Włodarek, 2019).

Alzheimer’s disease and PD are the most common causes of cognitive impairment in the elderly, both neurodegenerative diseases characterized by progressive memory loss and cognitive decline. AD, PD and all neurological diseases occur within a tissue separated from blood by the blood-brain barrier. Our understanding of this disease is still far from complete. The factors trigger this disease occurring may have a correlation with the individual’s resistance at many levels, such as intestinal flora colonization, 5-HT levels, etc. (Galts et al., 2019; Correia et al., 2021; Ou et al., 2021).

Research by Cirrito et al. (2011) shed light on utilizing selective serotonin reuptake inhibitor (SSRI) or directly augmenting the amount of extracellular 5-HT can apparently reduce the Aβ content in mouse brain tissue fluid by 25%. An increasing body of evidence has suggested that SSRI suppress 5-HT re-uptake, enhance the concentration of 5-HT in the brain, and diminish Aβ aggregation by reacting with receptors (Yao et al., 2020).

Holmqvist et al. (2014) injected α-Syn into the intestinal wall of rats and found that the protein was finally found in the brain, indicating that α-Syn could be spread from the intestinal neuron and microtubules associated with transportation to the brain, in turn PD occurs. The latest study, implanting gut flora of PD, AD patients and normal flora, respectively, into GF mice, and it was found that the former had motor deficits, weakened GI function and constipation, which further indicated that intestinal bacteria may be correlated to PD and AD diseases (Sampson et al., 2016). TP can penetrate the blood-brain barrier, thereby improving the cognitive impairment of the brain. EGCG has been shown to play a neuroprotective role in a series of cellular and animal models of neurological diseases. The spatial learning and memory ability of 5-HT gene deficient mice was impaired, and the plasma 5-HT concentration of stress mice with poor cognitive ability decreased significantly, while under the regulation of TP, the 5-HT concentration of stress mice increased and the cognitive function was improved (Chen et al., 2010).

Accordingly, modulating intestinal flora homeostasis, applying TP and SSRI to improve the concentration of 5-HT in the hippocampus of the brain, and weakening the accumulation of Aβ and α-Syn are expected to become a new therapeutic option for the management of neurological conditions (Mostert et al., 2008).

## Intestinal Flora-5-HT and Depression, Autism

Depression is an affective disorder that accompanied by symptoms of low emotions, inattention, together with decreased volition and behavior. It also includes symptoms like inappropriate suicidal thoughts, insomnia, and anorexia (Bair et al., 2003). Modern studies have shown that depression is directly related to 5-HT, and the production of modern antidepressants mainly focus on elevating 5-HT concentration in the brain or inhibiting 5-HT uptake by platelets (Morais et al., 2021). Oral L-theanine can increase the levels of 5-HT and dopamine in striatum, hypothalamus and hippocampus (Zhu et al., 2012). Abnormal structure of the intestinal flora induce inflammation with the production of various inflammatory factors, whereby entering the CNS through circulatory pathway, activating microglia in the glial cells, and promoting depressive episodes. Emerging evidence suggests that implantation the flora of depression and normal people into GF mice, respectively, showing the former had depression-like behaviors, flora diversity had changed and was highly similar to that of depressive patients (Zheng et al., 2016). MGB axis connects the emotions of the brain with the peripheral control and function of the intestine. 5-HT is a key element of this axis, acting as a neurotransmitter in the CNS and the ENS of the intestinal wall. *Lactobacillus* and *Bifidobacterium* are the two major probiotics, attenuating immune inflammatory factors, restoring the integrity of the intestinal barrier, regulating tryptophan metabolism, influencing 5-HT and mood.

The effect of 5-HT on mood has been studied through an acute tryptophan depletion technique whereby reducing dietary tryptophan leads to a lowering in brain 5-HT levels, which acquires analysis for 5-HT dependent behavior. The subjects ate food rich and lack in tryptophan, respectively, and then used pictures to exacerbate indignation of the subjects, ultimately utilized magnetic resonance imaging technology to observe the brain’s response (Alkhalaf and Ryan, 2015). The results show that when tryptophan-deficient causing a decrease in the level of 5-HT in the body, the anger responses derived from brain are uneasy to suppress, meanwhile the signaling communications between the frontal lobe of the brain and the amygdala are reduced (Okaty et al., 2019). The frontal lobe is responsible for controlling emotions such as anger, while the amygdala is anger-related, yielding the view when deficient in 5-HT, the rational frontal lobe is unable to control the angry amygdala (Récamier-Carballo et al., 2017).

Autism, is a common disease of infants and young children with congenital developmental disorders. After treatment with *Bacteroides fragilis* or *Bacteroides polymorpha*, the structure of autism model mice changed close to normal mice (Iovene et al., 2017), and their autism symptoms were improved. Recent research also demonstrates that *Lactobacillus* has the ability to maintain intestinal homeostasis and facilitate the therapeutics of autism (Sgritta et al., 2019). The antidepressant effect of TP may be due to its ability to block the reuptake of the neurotransmitter 5-HT by the presynaptic membrane of nerve endings and increase the concentration of monoamines in the synaptic space (Wei et al., 2015). In addition, TP inhibited the activity of monoamine oxidase B and increased the level of monoamine in rat C6 astrocytes in a dose-dependent manner, indicating that monoamines play an important role in the pathophysiology of depression (Zhu et al., 2012; Frolkis et al., 2019). All of these indicate that the colonization of intestinal flora impact host behavior and the occurrence of related diseases.

## Intestinal Flora-5-HT and Immune Disease Like Inflammatory Bowel Syndrome

Inflammatory bowel syndrome is a clinically common GI dysfunction disease along with manifestations such as abdominal pain, bloating, and changes in bowel habits, accompanied by anxiety, depression, irritability and other mental problems (Fond et al., 2014). These phenomena are considered to be hypersensitivity reactions of the MGB system. Studies have shown that the intestinal flora can elicit mononuclear macrophages and mast cells, release 5-HT, produce changes in IL-10, IL-6, IL-1β, TNF-α, and IFN-γ, and inhibit intestinal motility (Liu et al., 2009).

Experimental studies have shown that as the main body of ECs, mucosal mast cells are activated after acute stress, and they increase or approach the enteric nerve after chronic stress (Albert-Bayo et al., 2019). These mast cells release neuropeptides, namely 5-HT, proteases and pro-inflammatory cytokines, which are known to cause modifications in IBS intestinal sensory, motor, secretory and osmotic media. The amount of 5-HT in neonates increases after acute stress, while the increased 5-HT is closely related to the occurrence of IBS.

It is well known that stress-related can trigger alterations in intestinal motility, visceral sensitivity and intestinal secretion, as well as the occurrence of many extra-intestinal stress-related diseases (such as anxiety, depression or chronic pain syndrome), whereas 5-HT is a non-negligible therapeutic strategy in the treatment of stress-related diseases.

Usage of probiotics to ameliorate the intestinal environment of IBS and related mental problems may become an effective intervention. It is possible to renovate the diversity and stability of the intestinal flora as target, using intestinal prebiotics, antibiotics and fecal bacteria transplantation to elevate the abundance of symbiotic microorganisms, with decreasing flora ratio that can ameliorate IBS symptoms (such as *Clostridium*, *E. coli*, *Salmonella*, *Shigella*, and *Pseudomonas*), to achieve the purpose of treating IBS and other 5-HT-related MGB axis perturbations (Currò et al., 2017).

At present, studies have shown that there is a certain relationship between theanine and PPs in tea and 5-HT. TP not only produces neuroactive microbial metabolites, but also inhibits harmful bacteria, providing a source of nutrition for antidepressant-related probiotics. As a potential antidepressant, TP can treat inflammation in IBS, inhibit the activation of kynurenine pathway, promote digestive system and stop diarrhea (Mikocka-Walus et al., 2020; Cao et al., 2021). Under treatment such as antidepressants or psychotherapy, neurons may grow again. The study found that the level of brain-derived neurotrophic factor before neurogenesis increased with the treatment of antidepressants, and this increase seemed to be related to the degree of recovery of depression (Brunoni et al., 2008).

## Neuroprotective and Anti-Inflammatory Effects of Tea Polyphenols

Vascular dementia (VD) is a brain function disease with intellectual disability triggered by insufficient blood and oxygen supply to the brain owing to various diseases (Romay et al., 2019). It is the second largest dementia disease after AD. Hippocampus is a crucial structure that participates in the body’s spatial learning and memory. Cerebral ischemia and hypoxia can easily stimulate hippocampal neuronal apoptosis and decrease cognitive function. Several observational studies have indicated that after TP-intervention, the morphology of neurons in the CA1 area of the hippocampus of VD rats tended to be normal, and the deposition of Aβ decreased, illustrating that it has a protective effect on neurons (Simão et al., 2012; Libro et al., 2016; Fernando et al., 2017).

The PPs compounds may exert neuroprotective effects through anti-aggregation properties. TP inhibit the formation of wild-type α-Syn filamentous aggregates and depolymerize the fibrous α-Syn. EGCG can effectively restrain the formation of α-Syn filaments, and also convert large toxic α-Syn filaments into small non-toxic and non-fixed shape protein aggregates. Green tea intake can prevent the decrease of glutathione peroxidase, indicating that green tea has a protective effect on age-related oxidative damage. A wealth of evidence now implicates that TP is potentially bioactive substances with neuroprotective and neuromodulating activities (Wang Y. et al., 2021).

Studies have shown that interfere with EGCG in high-fat diet rats can significantly control the expression level of C-reactive protein, alleviate the red blood cell sedimentation rate and total white blood cell count, as well as inhibit the formation of atherosclerosis through anti-inflammatory effects (Song et al., 2021). In the insulin-resisting rat model, TP prevents the expression of inflammatory cytokines, enhance the production of anti-inflammatory proteins, and relieve the damage of chronic inflammation to the myocardium by regulating signaling communication associated with insulin, lipid metabolism and inflammation (Xia et al., 2019). In a LPS-induced rat model of acute lung injury, EGCG diminishes the expression of inflammatory factors by managing signal pathways, attenuates the accumulation of neutrophils in the lungs, and has a protective effect on lung injury (Wang M. et al., 2021).

While a small number of studies have reported the intake and brain distribution of edible TP, there are still great uncertainties in the dosage, absorption, metabolism, tissue distribution, and intracellular accumulation and excretion of TP (Hong et al., 2021). Therefore, these aspects may be the focus of future research.

## Conclusion

Tea polyphenols, especially EGCG has been the focus of research owing to it multiple protective effects on various diseases of the host. A wealth of epidemiological studies and clinical trials has shown that appropriate supplementation of TP has obvious protective effects on chronic neurological diseases and related immune diseases. As such the low bioavailability of oral administration of TP, clinical application meets many as-yet-uncharacterized challenges. It is still unclear how to effectively deliver EGCG to the target site. Increasing information from both clinical and preclinical fields presents convincing evidence that crosstalk between the gut microbiota and associated metabolism along with the mammalian nervous system shapes dysregulated and stable neural processes. As the vast metabolites among gut microbes, SCFAs and 5-HT are proposed as a kind of transmission media, which establishes the inextricable connection between brain neurons and enteric neurons. Therefore, the co-evolution of host with their related microbial communities and metabolites seems to have led to complex biological communication between the intestine and the brain. This is a fascinating prospect that requires more research in the future, but also provides a promising new way for the regulation of mental and neurological diseases.

## Author Contributions

MH: conceptualization, validation, and writing – original draft. LC and PZ: supervision and writing – original draft. YL: conceptualization and validation. ZW: writing – original draft. XZ: supervision and writing – review and editing. All authors contributed to the article and approved the submitted version.

## Conflict of Interest

The authors declare that the research was conducted in the absence of any commercial or financial relationships that could be construed as a potential conflict of interest.

## Publisher’s Note

All claims expressed in this article are solely those of the authors and do not necessarily represent those of their affiliated organizations, or those of the publisher, the editors and the reviewers. Any product that may be evaluated in this article, or claim that may be made by its manufacturer, is not guaranteed or endorsed by the publisher.
